# South West Urological Oncology Group Autumn Meeting 1991

**Published:** 1992-12

**Authors:** 


### South West Urological Oncology Group

### Meeting on Invasive Bladder Cancer, October 1991

The South West Urological Oncology Group was established by Gary Sibley (Consultant Urologist) and Hugh Newman (Consultant Clinical Oncologist) in Spring 1991 to encourage a multi-disciplinary approach to the management of urological tumours. In addition to providing a forum for discussion of current clinical practice, the society aims to encourage research and participation in clinical trials in order to improve the quality of care within the region for this important group of patients. Links have been established with the Regional Cancer Organisation to help co-ordinate these aims.

The inaugural meeting was held at the Bristol Royal Infirmary in Spring 1991 on the subject of prostate cancer; participants included urologists, oncologists, radiologists and pathologists. It is planned to hold future meetings in the Spring and Autumn each year, and abstracts from the Autumn Meeting 1991 are published here. This was held at the Bristol Royal Infirmary on 2nd October 1991, with Mr. R. Hall from Newcastle attending as the guest speaker.

### SURGICAL ASPECTS IN THE MANAGEMENT OF INVASIVE BLADDER CANCER

G.N.A. Sibley
Department of Urology, Bristol Royal Infirmary

Cystectomy for invasive bladder cancer (either alone or following pre-operative radiotherapy) is associated with higher survival rates than radiotherapy alone, especially for patients under the age of 65. However, one of the factors rendering surgery less popular in the past was the need for simultaneous urinary diversion (by ileal conduit or uretero-sigmoidostomy).

With developments in reconstructive surgical techniques, urinary diversion may now be avoided in selected cases by construction of a "neo-bladder" from the intestine that is then anastomosed directly to the membranous urethra. The intestinal "bladder" may be constructed from ileum, the sigmoid colon or using an ileo-caecal segment. The continent reservoir produced is emptied by abdominal straining, although intermittent self-catheterisation may be needed to achieve complete emptying. The technique is applicable mainly for male patients, in whom potency may also be retained by preservation of the neurovascular bundles innervating the corpora cavernosa. It is contraindicated in patients with multifocal tumours or widespread carcinoma in situ due to the risks of urethral recurrence.

When anastomosis to the membranous urethra is not feasible (in women and in all patients where the urethra must be removed), the bowel reservoir can be given a continent catheterisable stoma instead. The continence mechanism can be provided by submucosal tunnelling of a narrow tube, such as appendix or ureter, into the reservoir (Mitrofanoff principle), or by intussusception of a length of ileum to form a non-return valve (the Kock pouch).

The use of these reconstructive surgical techniques offers a much improved quality of life for selected patients requiring cystectomy for invasive bladder cancer.

### "RAPID RADIOTHERAPY RAISES HOPES". A PLACE FOR ACCELERATED RADIOTHERAPY?

H. Newman
Department of Clinical Oncology
Regional Oncology Centre, Bristol

There is evidence to suggest that transitional cell carcinoma of the bladder has the ability to repopulate rapidly, and therefore may be more effectively dealt with by accelerated radiotherapy. The Co-operative Urological Oncology Group has been conducting a randomised trial comparing conventionally fractionated radiotherapy (64 Gy in 32 fractions in 6.5 weeks) against accelerated treatment. Two dose-levels were used in the accelerated arm: 64 Gy in 32 fractions in 28 days, treating twice daily but with a short gap after week 1, and 57.5 Gy, with the same schedule (i.e. 2 Gy or 1.8 Gy per fraction respectively).

Eighteen patients have been entered from the satellite radiotherapy unit in Bath, and randomisation was equal between accelerated and conventional treatments. Without prejudice to the ultimate report of the Working Party, the initial experience was reviewed, with a minimum follow-up of 12 months from randomisation.

The acute tolerance of the accelerated regimen was comparable to conventionally treated patients, with no obvious increase in bladder, small bowel or rectal morbidity. There has been no obvious excess of late-occurring complications, though one patient in the accelerated arm has required permanent catheterisation because of a contracted bladder. No definitive survival or local control data can be given, but local control appears to be similar in the two groups.

### SYSTEMIC CHEMOTHERAPY FOR ADVANCED BLADDER CANCER

R. Hall
Department of Urology
Freeman Hospital, Newcastle

Is systemic chemotherapy of any value in the routine management of advanced bladder cancer? This is a reasonable question to which we have a few answers.

1. For patients with T4b tumours or metastatic bladder cancer chemotherapy offers worthwhile palliation of bladder symptoms, pelvic or bone pain, lymphoedema, breathlessness and other symptoms. Overall survival is not increased more than a few months although a few long term survivors have been reported. If palliation is the main benefit, outpatient chemotherapy rather than inpatient treatment would be an advantage. For this reason the MRC is conducting a trial of Methotrexate/Vinblastine as outpatient treatment compared with CMV.

2. Chemotherapy can be successful in downstaging inoperable T3b or T4b tumours so that they may be salvaged by cystectomy. Whether these patients live any longer as a result of this treatment is not yet known.

3. Results from 104 patients treated in Newcastle show that 19% of T3-T4b patients, having achieved complete remission with Cisplatin containing combination chemotherapy, survive disease free at 3 years with no treatment other than chemotherapy - no TUR, no cystectomy, no radiotherapy. These results indicate significant chemotherapeutic activity, but suggest that chemotherapy used alone is inadequate as definitive treatment. Tumour sensitivity to chemotherapy overlaps radiosensitivity: a 32% initial CR rate with chemotherapy was increased to 49% by "salvage" full dose radiotherapy.

4. The benefit of adjuvant chemotherapy is still uncertain, but the MRC/EORTC trial is progressing well with 430 patients randomised from 16 countries. A total of 900 patients is needed, and recruitment is via the MRC Cancer Trials Office in Cambridge.

### MRC/EORTC TRIAL OF NEOADJUVANT CHEMOTHERAPY - THE INITIAL BRISTOL EXPERIENCE

H. Newman
Department of Clinical Oncology
Regional Oncology Centre, Bristol

The MRC/EORTC neoadjuvant chemotherapy trial randomises patients either to receive 3 cycles of Cisplatin, Methotrexate and Vinblastine before local radical treatment to the bladder, or to proceed straight to local treatment.

To October 1991 the Bristol group had entered 6 patients, of whom 4 had been randomised to chemotherapy. In 2 patients radical surgery was the local treatment because of tumour extent or urethral involvement; the remainder received radiotherapy. The Bristol experience was reviewed in general terms, without prejudice to the ultimate report of the Working Party, with a minimum follow-up of 10 months from randomisation.

In terms of general acute tolerance, all patients had mild toxicity from chemotherapy. General malaise was experienced by all patients, though this recovered a month or so afterwards. Anaemia was a problem in 2 patients, with a significant fall in haemoglobin after the third cycle, necessitating transfusion before the post-chemotherapy biopsy. One patient required the omission of Cisplatin after the first cycle because of nephrotoxicity. All patients received the elective folinic acid rescue with all cycles, and there were no other significant dose modifications or delays, and no episodes of febrile neutropenia. Post-chemotherapy biopsies were done uneventfully, but results were difficult to interpret as biopsied patients had T2 lesions resected initially. There were no additional problems with radiotherapy in the neoadjuvant group, though one patient who had only pre-operative radiotherapy and elective cystectomy developed some rectal morbidity 6 months later, presumably mainly due to radiation in view of the timing. One neoadjuvant primarily-irradiated patient required salvage cystectomy, and had node-positive disease.

In summary, this rather demanding protocol was, in our initial experience, quite well tolerated by the patients, especially as 3 of them were over 70 years old. We are continuing to enter patients into this trial.

### CYTOGENETIC ANALYSIS OF BLADDER TUMOURS AS A PREDICTOR OF CLINICAL OUTCOME

R. Persad
Department of Urology
Bristol Royal Infirmary

Prediction of disease recurrence or progression in patients with transitional cell carcinoma (TCC) of the bladder is difficult. Chromosomal markers have been noted in association with TCC and this study examined the prognostic value of bladder tumour karyotyping.

Eighty-five newly diagnosed cases of bladder TCC were assessed, together with a control group of 10 patients with normal bladder biopsies taken at cystoscopy for haematuria. A short-term culture technique was employed and colchicine used to arrest cell growth in metaphase. Standard cytogenetic techniques were used and the following parameters observed: rate of growth culture, average number of metaphases, modal chromosome number, and the presence of marker chromosomes.

The control group all had normal karyotypes with no obvious chromosomal markers present. The modal number was 45-46 and the mean rate of growth to harvesting was 8 days. Culture yield was 62% for invasive tumours and 76% for superficial tumours, superficial papillary tumours growing faster in culture than solid invasive ones (2 days vs. 6 days). The mean modal number for invasive tumours was 63, compared with 56 for superficial tumours. All the aggressive (G3) invasive tumours had more than 3 markers present: changes included trisomy of 7, monosomy of 9 and translocations of various chromosomal fragments onto the short arm of chromosome 1.

Mean clinical follow-up was 19 months (9-90), and during this period 35% of tumours recurred and 18% progressed. Recurrence and progression were both associated with a significantly higher modal number and frequency of chromosomal abnormalities than non-recurrence. Ten (77%) of the superficial low grade tumours with markers recurred, 7 with progression.

The following papers were also given:

### PATHOLOGICAL INDICATORS OF MALIGNANT POTENTIAL

C. Collins
Department of Histopathology
Bristol Royal Infirmary

### BIOLOGICAL ASPECTS OF ACCELERATED RADIOTHERAPY

T. Sheehan
Department of Clinical Oncology
Regional Oncology Centre Bristol

### CHEMOTHERAPY IN METASTATIC UROTHELIAL CANCER

C. Tyrell
Department of Clinical Oncology
Freedom Fields Hospital Plymouth

